# Descriptive epidemiology of stigma against depression in a general population sample in Alberta

**DOI:** 10.1186/1471-244X-10-29

**Published:** 2010-04-19

**Authors:** Trevor M Cook, JianLi Wang

**Affiliations:** 1 Department of Community Health Sciences, Faculty of Medicine, University of Calgary, 3330 Hospital Drive NW, Calgary, Canada; 2 Department of Psychiatry, Faculty of Medicine, University of Calgary, 3330 Hospital Drive NW, Calgary, Canada

## Abstract

**Background:**

Mental health illnesses, such as depression, are responsible for a growing disease burden worldwide. Unfortunately, effective treatment is often impeded by stigmatizing attitudes of other individuals, which have been found to lead to a number of negative consequences including reduced help-seeking behavior and increased social distance. Despite the high prevalence of depression in Canada, little research has been conducted to examine stigma against depression in the Canadian general population. Such information is crucial to understanding the current state of stigmatizing attitudes in the Canadian communities, and framing future stigma reduction initiatives. The objectives of this study were to estimate the percentages of various stigmatizing attitudes toward depression in a general population sample and to compare the percentages by demographics and socioeconomic characteristics.

**Methods:**

We conducted a cross-sectional telephone survey in Alberta, Canada, between February and June 2006. Random digit dialing was used to recruit participants who were aged 18-74 years old (n = 3047). Participants were presented a case vignette describing a depressed individual, and responded to a 9-item Personal Stigma questionnaire. The percentages of stigmatizing attitudes were estimated and compared by demographic and socioeconomic variables.

**Results:**

Among the participants, 45.9% endorsed that depressed individuals were unpredictable and 21.9% held the view that people with depression were dangerous. Significant differences in stigmatizing attitudes were found by gender, age, education, and immigration status. A greater proportion of men than women held stigmatizing views on each stigma item. No consistent trend emerged by age in stigma against depression. Participants with higher levels of education reported less stigmatizing attitudes than those with less education. Participants who were not born in Canada were more likely to hold stigmatizing attitudes than those who were born in Canada.

**Conclusion:**

In the general population, stigmatizing attitudes towards depression differ by demographic characteristics. Men, those with less education and immigrants should be the targets of stigma reduction campaigns.

## Background

Major depression is a prevalent mental disorder in the general population and is a leading cause of disease burden [1]. The annual prevalence of major depression in Canada and in the United States was 4.8% and 6.8% in 2002, respectively [2,3]. To reduce the disease burden, comprehensive interventional strategies including primary and secondary prevention are needed. However, these efforts are often impeded by stigma against mental illness. Stigma towards mental illness may negatively affect individuals' willingness to seek help [4-6]. Other consequences of discrimination against people with mental illness include social distancing and exclusion [7,8], exacerbation of patient burden caused by the illness [9], chronic social impairment [10], and reduced life satisfaction [8,11]. Thus, one of the mandates of the Mental Health Commission of Canada is to conduct a national campaign to reduce stigma against mental illness [12]. Despite the high prevalence of major depression in the general population, stigmatizing attitudes towards depression in the general population are not well studied. Moreover, there is a lack of descriptive information about stigma against depression. Such information is critical to our understanding about the current status of stigma in the community, and providing a basis for mental health promotion and stigma reduction initiatives.

Mental health research has revealed three types of stigma: self-stigma - one's response to their own mental illness [5,13]; personal stigma - one's attitude towards a person with mental illness; and perceived stigma - one's belief about another's attitudes toward a person with mental illness [13]. All three types of stigma should be the targets of anti-stigma campaigns. To facilitate the development of anti-stigma programs targeting the general population, our study focused on personal stigma against depression.

Previous studies have found that depression stigma varies across demographic groups [14,15]. Further, mental health stigma has been found to have strong cultural roots and strong cross-cultural variations in its prevalence [16-18]. One Australian study found that in adults aged 18 years and over, the proportions of people holding views of personal stigma against depression were significantly higher among men, those with less education, and those born overseas, while age was positively associated with depression stigma in linear regression models [14]. A qualitative Australian study found participants reporting high degrees of depression stigma when individuals were viewed to be responsible for their own mental illness, a threat, or undesirable company [19].

In Canada, the study conducted by Wang and colleagues [15,20] found that men had a lower level of depression literacy [20] and were more likely to hold stigmatizing attitudes than women [15,21]. A higher level of education and being a health professional were negatively associated with depression stigma. In the Australian and the Canadian studies [14,15], the same Depression Stigma Scale, which is a dimensional scale, was used. As there is not a meaningful cutoff for the depression stigma scores, factors associated with the depression stigma scores were examined in linear regression modeling [14,15]. However, the beta coefficients in linear regression models are mathematical values and may not reflect important changes from clinical and public health perspectives. For example, what does a one or two point changes in the beta coefficient mean, and does the changes have significant meanings from the clinical and population health perspectives? In current analysis, we examined specific stigmatizing attitudes by demographic characteristics, providing more interpretable descriptive results about stigma against depression in the general population.

The objectives of this analysis were to (1) estimate the percentages of various stigmatizing attitudes towards depression in a general population sample, and (2) estimate and compare the percentages of various stigmatizing attitudes by demographic and socioeconomic characteristics.

## Methods

### Study Population and Sampling

From February to June 2006, we conducted a cross-sectional study examining depression literacy and stigma in Alberta, Canada. The target population was household residents in Alberta, aged 18 - 74 years old. Participants were recruited using random digit dialing method. Data was collected by interviewers of the Survey Unit of the Calgary Health Region (now Alberta Health Services), using the method of computer assisted telephone interview. Detailed information about sampling procedures can be found in previous publications [15,20]. This study was approved by the Conjoint Health Research Ethics Board of the University of Calgary. The final sample consisted of 3084 participants (response rate at the individual level = 75.2%). Among the participants, 37 participants were excluded from this analysis as their ages did not fall between the study requirements (aged 18 - 74 years), likely due to data entry errors. In this analysis, 3047 participants were included.

### Depression Literacy Case Vignette

In this study, we first presented a case vignette depicting a person (John or Mary) with major depression [15,20]. The case vignette is as follows:

"John is 30 years old. He has been feeling unusually sad and miserable for the last few weeks. Even though he is tired all the time, he has trouble sleeping nearly every night. John also doesn't feel like eating and has lost weight. He cannot keep his mind on his work and puts off making any decisions. Even day-to-day tasks seem too much for him. This has come to the attention of John's boss who is concerned about his lowered productivity."

After the case vignette, participants were asked "what would you say, if anything is wrong with John/Mary?" We used the answers to this question to determine whether participants could recognize depression. In the survey, we randomly used the name "John" and "Mary" to minimize potential bias related to gender of the person in the case vignette. Preliminary analysis revealed no significant difference in responses based on the name of the person depicted in the case vignette.

### Personal Stigma

We administered a 9-item personal depression stigma scale, reflecting the personal attitudes towards John or Mary. This scale was developed by Griffiths and colleagues [22]. For each question in our study (and the original scale), respondents answered using a 5-point Likert scale - strongly agree, agree, neither agree nor disagree, disagree, and strongly disagree. The depression stigma scale in our study yielded a Cronbach's alpha of 0.715, which was close to that of Griffiths *et al*. (alpha = 0.76) [22]. In our analysis, we combined "strongly agree" and "agree" for each item to indicate the presence of personal stigma [13]. Additionally, we summed the score of each item to derive a total stigma score. In our study, the total stigma scores ranged from 0 to 34, with a higher score indicating a higher level of stigma.

### Demographic and Socioeconomic Variables

Demographic and socioeconomic data was collected on all participants, including gender, education, age, employment status, immigration status, income, marital status, areas of residence (urban or rural), and whether or not participants were a health professional, or mental health professional. We classified participants into four groups by age (18-24 years old, 25 - 54 years old, 55 - 64 years old, 65 - 74 years old). These categories are commonly used in psychiatric epidemiological studies. The age categorization was based on the facts that people of age 18 and 24 years old are considered young adults; the ages from 25 to 54 years are adulthood; between 55 and 64 years, biological changes are prominent, especially for women; those aged 65 and over are considered seniors. Education was split into three groups based on educational institution attended: (1) attended or completed high school, (2) attended or completed college, and (3) attended or completed university or higher education. Employment status was determined as whether or not the respondent had worked in a job or business in the previous week. Immigration status was determined by their self report of whether or not they were born in Canada. Annual personal income was split into four groups: (1) Those with an annual income less than $30 000, (2) those with an annual income between $30 000 and $60 000, (3) those who earned $60 000 - $80 000 annually and (4) those who earned more than $80 000 annually. As personal income is a sensitive issue, we did not ask for the exact annual income, rather we asked in which of the previously described income groups their income would fit. Marital status was classified as (1) married or common-law, (2) single and never married, and (3) divorced, separated, or widowed. We considered participants' area of residence as urban area if they resided, worked, or were attending school in Calgary or Edmonton (urban), or rural area if they lived, worked, or attended school elsewhere in Alberta.

### Analysis

The percentages of stigmatizing attitudes of the 9-item stigma scale were estimated. The percentages were then compared by demographic and socioeconomic characteristics using Chi square (χ^2^) tests. We conducted multivariate linear regression modeling to examine the relationships between the demographic and socioeconomic variables and total stigma scores. We first examined possible effect modifications by gender and other variables. If an effect modification was found, the associations between selected variables and stigma scores were estimated separately in men and women. The analyses were weighted to account for the effects of differential sampling probability, household size, number of telephone line and gender-age distribution of the general population in Alberta. As we compared the percentages for 9 different items in the bivariate analysis, we set the significance level at 0.005. The analysis was conducted using STATA 10.0 [23].

## Results

The weighted and un-weighted demographic and socioeconomic characteristics of the participants can be found in previous publications [15,20]. The overall and gender specific percentages of various stigmatizing attitudes towards depression are presented in Table 1. Overall, unpredictability emerged as the most prevalent stigmatizing view of depression, with 45.9% of participants reporting that they believed the person with depression in the case vignette to be unpredictable. This was followed by the refusal to vote for depressed individuals (39.5%), not wishing to employ individuals suffering from depression (22.1%), depressed individuals being dangerous (21.9%), that people with depression could "snap out of it" if they wanted (16.7%), and that they would not tell others of their depression (13.6%).

**Table 1:** Percentages of various stigmatizing attitudes overall and by gender*

Stigma Item	Overall n = 2987	Gender (Weighted %)		*χ*^2^*(1)*	*P *=
		Male n = 1525	Female n = 1462		
People with a problem like (x)'s could snap out of it, if they wanted."	17.00	23.4	10.3	90.48	<0.001
"A problem like (x)'s is a sign of personal weakness..."	9.8	13.6	5.9	50.64	<0.001
"(x)'s problem is not a real medical illness..."	8.5	11.3	5.7	29.64	<0.001
"People with a problem like (x)'s are dangerous."	21.9	22.2	21.5	0.23	n/s
"It is best to a avoid people with a problem like (x)'s..."	3.2	4.8	1.4	29.08	<0.001
"People with a problem like (x)'s are unpredictable..."	45.9	57.8	42.2	15.63	<0.005
"If I had a problem like (x)'s, I would not tell anyone."	13.6	16.1	11.1	16.07	<0.001
"I would not employ someone if I knew they had a problem like (x)'s."	22.1	29.1	14.7	87.68	<0.001
"I would not vote for a politician if I knew they had a problem like (x)'s."	39.5	48.4	30.2	102.02	<0.001

*Note: The responses "Strongly Agree" and "Agree" have been combined to indicate the presence of stigma. All chi square tests had one degree of freedom. n/s = not significant

Men reported higher proportions of stigmatizing attitudes than women on all items, except in their views of the depressed person as dangerous. In some stigmatizing attitudes, such as whether or not John or Mary should be avoided, the difference between men and women was only 3.4%, while 18.2% more of men than women reported that they would not vote for a politician if they knew the person was depressed (48.4% versus 30.2%, χ^2 ^(1) = 102.02, p < 0.001). Men were more than twice as likely as women to believe that individuals suffering from depression could "snap out of it" (23.4% to 10.3%, χ^2 ^(1) = 90.48, p < 0.001) or should be avoided (4.8% to 1.4%, χ^2 ^(1) = 29.08, p < 0.001). It is also worth noting that over half (57.8%) of male respondents reported that depressed individuals were unpredictable, compared to 42.2% of female participants (χ^2 ^(1) = 15.63, p < 0.005).

Table 2 contains age specific percentages of stigmatizing attitudes among the participants. As seen from the table, the trends were not consistent across items. When asked if depressed individuals could "snap out" of their illness, the percentages of stigmatizing attitude decreased with age (χ^2 ^(3) = 28.17, p < 0.005). Conversely, when asked if they would not vote for a politician if they knew the person had depression, the percentages increased with age (χ^2 ^(3) = 35.53, p < 0.001). With respect to whether depression was a real medical illness, those over 65 and under 24 years old were more likely to endorse that depression was not a real illness, compared to those aged 25-64 (15% & 12.1% vs. 7.0%. χ^2 ^(3) = 26.75, p < 0.005).

**Table 2:** Percentages of various stigmatizing attitudes by age*

	Age (Weighted %)				*X*^2^*(3)*	*P *=
Stigma Item	18-24 n = 548	25-54 n = 1203	55-64 n = 1024	65-74 n = 211		
People with a problem like (x)'s could snap out of it, if they wanted."	22.0	18.9	12.6	14.0	28.17	<0.005
"A problem like (x)'s is a sign of personal weakness..."	11.5	10.6	7.2	13.7	14.29	n/s
"(x)'s problem is not a real medical illness..."	12.1	7.0	7.0	15.0	26.75	<0.005
"People with a problem like (x)'s are dangerous."	17.8	23.9	20.7	26.4	11.45	n/s
"It is best to a avoid people with a problem like (x)'s..."	5.2	2.7	2.4	4.3	11.48	n/s
"People with a problem like (x)'s are unpredictable..."	45.8	43.5	47.4	52.4	7.31	n/s
"If I had a problem like (x)'s, I would not tell anyone."	10.4	14.5	13.6	17.1	7.96	n/s
"I would not employ someone if I knew they had a problem like (x)'s."	23.8	18.9	23.9	27.7	13.33	n/s
"I would not vote for a politician if I knew they had a problem like (x)'s."	32.7	37.5	42.6	54.4	35.53	<0.001

*Note: The responses "Strongly Agree" and "Agree" have been combined to indicate the presence of stigma. All chi square tests had three degrees of freedom. n/s = not significant.

The estimated percentages of stigmatizing attitudes by educational levels are in Table 3. Significant differences by educational levels were found in 5 of 9 stigma-related questions. Participants who were at the higher educational level were less likely to report that "X could snap out of it" (13.0% versus 22.8%, χ^2 ^(2) = 37.15, p < 0.001), "a problem like X's is a sign of personal weakness" (6.8% versus 13.0%, χ^2 ^(2) = 21.84, P < 0.005), "X's problem is not a real medical illness" (5.9% versus 13.5%, χ^2 ^(2) = 47.25, p < 0.001), "People with a problem like X's are dangerous" (14.9% versus 27.8%, χ^2 ^(2) = 48.45, p < 0.001) and "People with a problem like X's are unpredictable" (38.7% versus 53.0%, χ^2 ^(2) = 40.23, p < 0.001). Educational levels were not related to other stigmatizing attitudes.

**Table 3:** Percentages of various stigmatizing attitudes by educational levels*

	Education (Weighted %)			*χ*^2^*(2)*	*P *=
Stigma Item	High School or less n = 971	College or Technical School attended n = 1002	University Attended n = 1006		
People with a problem like (x)'s could snap out of it, if they wanted."	22.8	15.3	13.0	37.15	<0.001
"A problem like (x)'s is a sign of personal weakness..."	13.0	9.6	6.8	21.84	<0.005
"(x)'s problem is not a real medical illness..."	13.5	6.1	5.9	47.25	<0.001
"People with a problem like (x)'s are dangerous."	27.8	23.1	14.9	48.45	<0.001
"It is best to a avoid people with a problem like (x)'s..."	4.4	2.8	2.3	8.10	n/s
"People with a problem like (x)'s are unpredictable..."	53.0	46.1	38.7	40.23	<0.001
"If I had a problem like (x)'s, I would not tell anyone."	13.3	13.3	14.2	0.45	n/s
"I would not employ someone if I knew they had a problem like (x)'s."	24.9	21.1	20.5	6.39	n/s
"I would not vote for a politician if I knew they had a problem like (x)'s."	43.1	40.3	35.1	13.45	n/s

*Note: The responses "Strongly Agree" and "Agree" have been combined to indicate the presence of stigma. All chi square tests had two degrees of freedom. n/s = not significant.

Participants who were not born in Canada were more likely to report stigmatizing attitudes than those who were born in Canada on 5 out of 9 questions (see Table 4). When compared to individuals born in Canada, individuals not born in Canada were more likely to endorse that individuals could "snap out" of their depression (29.6% vs. 15.1%, χ^2 ^(1) = 50.12, p < 0.001); perceive depression as a sign of personal weakness (26.0% vs. 7.3%, χ^2 ^(1) = 136.11, p < 0.001); or believe it best to avoid individuals with depression (9.0% vs. 2.3%, χ^2^(1) = 50.97, p < 0.001). Individuals born outside Canada were more likely to believe that depression was not a real medical illness (17.6% vs. 7.2%, χ^2 ^(1) = 46.65, p < 0.001), or that they would not vote for a candidate they knew to be depressed (47.5% vs. 38.3%, χ^2 ^(1) = 11.91, p < 0.005). The two groups were not significantly different in other stigmatizing attitudes (unpredictability, danger, employment, and notification of illness).

**Table 4:** Percentages of various stigmatizing attitudes by immigration status*

	Immigration Status (Weighted %)		*χ*^2^*(1)*	*P *=
Stigma Item	Born in Canada n = 2599	Not Born in Canada n = 387		
People with a problem like (x)'s could snap out of it, if they wanted."	15.1	29.6	50.12	<0.001
"A problem like (x)'s is a sign of personal weakness..."	7.3	26.0	136.11	<0.001
"(x)'s problem is not a real medical illness..."	7.2	17.6	46.65	<0.001
"People with a problem like (x)'s are dangerous."	21.7	22.7	0.19	n/s
"It is best to a avoid people with a problem like (x)'s..."	2.3	9.0	50.97	<0.001
"People with a problem like (x)'s are unpredictable..."	45.3	46.4	2.24	n/s
"If I had a problem like (x)'s, I would not tell anyone."	14.0	11.5	1.81	n/s
"I would not employ someone if I knew they had a problem like (x)'s."	21.6	26.0	3.70	n/s
"I would not vote for a politician if I knew they had a problem like (x)'s."	38.3	47.5	11.91	<0.005

*Note: The responses "Strongly Agree" and "Agree" have been combined to indicate the presence of stigma. All chi square tests had one degree of freedom. n/s = not significant.

Neither employment status (working or not working), nor whether participants lived in a rural or urban setting were found to have significant differences with respect to the stigmatizing attitudes in bivariate analysis. Results are available upon request.

We found that participants with an annual income of $80,000 or more were more likely to indicate they would not vote for an individual if they knew them to be depressed than those with an annual income below $30,000 (46.5% vs. 33.5%, χ^2 ^(3) = 23.41, p < 0.005). Participants who were married or in a common-law relationship (41.3%) and those who were divorced, separated or widowed (43.8%) indicated that they would not vote for a candidate with depression (χ^2 ^(2) = 16.87, p = 0.005). Only 33.1% of those who were single or never-married expressed a similar attitude. Marital status and income were not found to be correlated with other stigmatizing attitudes.

Among health professionals, 29.2% indicated that they would not vote for a politician if they knew they were depressed, compared to 40.5% of those who were not health professionals (χ^2 ^(1) = 12.91, p < 0.005). Mental health professionals were less likely than non-mental health professionals to withhold their condition from others (2.9% versus 13.8%) (χ^2 ^(1) = 5.76, p < 0.005).

In multivariate linear regression modeling (F = 28.69, p < 0.001), we found effect modifications between gender and case recognition (β = -1.17, standard error = 0.57, p = 0.04) and between gender and immigration status (β = 1.59, standard error = 0.74, p = 0.03). This indicated that, the relationship of gender on stigma scores was modified by case recognition, and by immigration status. Women participants who could recognize depression in the case vignette were more likely to have lower stigma scores, while women who were immigrants were likely to have higher stigma scores. As such, multivariate linear regression models were conducted in men and in women separately. The results of the multivariate linear regression modeling are in Table 5.

**Table 5:** Results of multivariate linear regression modeling of stigma, overall and by gender

	Overall		Male		Female	
	β	SE	β	SE	β	SE
Age	-0.01	0.01	-0.02	0.01	-0.00	0.01
Gender	-1.11^+^	0.52	-	-	-	-
Immigration Status	1.71*	0.39	1.27^+^	0.56	2.45*	0.49
Income	0.19	0.11	0.40^+^	0.18	-0.19	0.14
Education	-0.96*	0.14	-1.06*	0.22	-0.85*	0.17
Health Professional Status	-1.10*	0.34	-1.17	0.83	-1.11*	0.35
Marital Status	0.12	0.16	-0.05	0.28	0.21	0.17
Employment Status	-0.14	0.25	-0.86	0.43	0.27	0.29
Rural/Urban residence	-0.45	0.23	-0.76^+^	0.35	-0.11	0.28
Case Recognition	-1.67*	0.57	-1.67*	0.39	-2.76*	0.42
						
**Regression Model Significance**						
Overall:	F = 28.69	P < 0.001	R^2 ^= 0.1602		df = 11, 2367	
Male:	F = 8.06	P < 0.001	R^2 ^= 0.0940		df = 9, 928	
Female:	F = 13.63	P < 0.001	R^2 ^= 0.1043		df = 9, 1431	

* p < 0.005, ^+ ^p < 0.05. SE = Standard Error.

Gender specific regression modeling (F = 8.06, p < 0.001 in men, F = 13.63, p < 0.001 in women) showed that immigration status and income levels were positively associated with stigma in men; while educational levels, rural/urban residence and case recognition were negatively associated with stigma scores in men (Table 5). In women, immigration status was positively associated with stigma; educational levels, being a health professional and case recognition were negatively associated with stigma. This indicated that, while male and female immigrants were more likely to have high stigma scores than non-immigrants, the effect was more pronounced in women. Men with a higher income were more likely to have high stigma scores. Individuals with a higher level of education were less likely to have high stigma scores. Females who were health professionals were less likely to have high stigma scores. Men who lived in an urban setting were less likely to have high stigma scores. For both men and women the ability to recognize depression was associated with lower stigma scores, and this effect was more pronounced in women than men.

## Discussion

This analysis provided descriptive information about personal stigma against depression in the general population of Alberta. One of the key findings was that 45.9% reported that "People with a problem like (x)'s are unpredictable" and 21.9% endorsed "People with a problem like (x)'s are dangerous." We also found significant differences in stigma against depression by gender, age, educational levels and immigration status. The associations between case recognition, immigration status and stigma scores were stronger in women than in men.

Dangerousness, avoidance and character weakness are the main elements in the mechanism underlying stigma against mental illness [4]. These attitudes were reported by 21.9%, 9.8%, and 3.2% of the participants respectively. It was unexpected that significant proportions of the participants held the views that "problem like John/Mary's is dangerous" and "persons with depression are unpredictable." These attitudes did not differ by age, however unpredictability differed by gender, and both differed by education. More studies are needed to investigate why the general public perceives people with depression as being dangerous and unpredictable. Results of such studies could have significant implications for stigma reduction.

Gender emerged as a significant factor associated with depression stigma. This was consistent with previous research indicating men had held higher stigmatizing attitudes than women [21]. Previous research has indicated that women have higher levels of depression literacy than men [15], and that increasing levels of mental health literacy is correlated with lower levels of depression stigma [20-22], therefore, the gender differences in the stigmatizing attitudes observed in this study were expected. This may also be due to observations that when compared to men, women are more likely to be exposed to depression [15], are three times more likely to experience a major depressive episode in response to certain events [24], and are twice as likely to be depressed [25] - potentially due to different gender-related risk factors [26], and emotional experience and response [27]. Research in other mental illnesses has found contrary results however - a German study found woman to have higher stigmatizing attitudes than men when dealing with schizophrenic individuals [28], while others have found no gender difference at all in dealing with depression [29]. As a result, future research into mental health stigma should continue in order to gain insight into mental health literacy and search for potential influences on gender differences, where they exist.

Age differences existed in three of the nine items of depression stigma and three different trends emerged. This difference in trends makes it difficult to allow conclusions to be drawn regarding the influence of age on depression stigma. A study by Wolkenstein and Meyer [30] points to the potential impact of 'political correctness' on mental health attitudes, and the impact of this phenomenon on perceived social desirability [30]. It is reasonable to assume that attitudes of political correctness and perceptions of social desirability differ by age groups, in addition to education background. Future research into stigma against mental illness should therefore address both mental health literacy, as well as perceptions of social desirability.

Participants who were at the higher educational level were less likely to report stigmatizing attitudes than others, which was consistent with the Australian study [14]. Over half (53.0%) of individuals who had only attended high school believed depressed individuals to be unpredictable, compared to only 38.7% of those who had attended University. When asked if these individuals were dangerous, the proportions were 27.8% to 14.9% for high school and university attendees respectively. The differences by educational levels suggest that in order to reduce fear in the general population towards individuals with depression, the messages may need to be tailored by people's educational levels. Nevertheless, the implications of the differences by educational levels need to be further investigated.

Our results found immigration status to be a major demographic variable in the examination of depression stigma. On certain stigma items, individuals not born in Canada were twice, three times, and in one case four times more likely to hold a stigmatizing attitude than those born in Canada. This is consistent with an Australian study that also found immigrants to hold higher stigmatizing attitudes [14]. A number of studies have also explored the impact of culture on depression stigma: Depression stigma has been found to be higher among Chinese Americans than White Americans [17], among White Americans than African Americans [16], and among older Korean Americans than younger Korean Americans [18]. These attitudes have been attributed to perceptions of family shame in Koreans [18], and a depression diagnosis as being "morally unacceptable" among Chinese Americans [31]. Further, it has been found that different cultural groups often experience different symptoms of depression, which may not only complicate diagnosis and mental health literacy, but also contribute to stigma against depression [31]. This is of particular concern given findings that the effectiveness of educational interventions is moderated and dependent upon an individual's beliefs about depression and its causation [32].

An unexpected result was the lack of difference in stigma between health and mental health professionals and the general public in most of the 9 items. Mental health professionals were found to differ only in their willingness to disclose their depression, and health professionals only in their willingness to vote for a depressed individual when compared to the general population. Health professionals are the group that mental health consumers deal with first when they seek health services. Therefore, reducing stigma in health professionals is critical. More research should be undertaken to further examine these relationships, as the ability of our analysis to draw meaningful calculations from these professional groups was limited by the small sample size of health professionals (n = 348) and mental health professionals (n = 74) in our study.

Case recognition emerged as a significant factor in stigma against depression in linear regression modeling. Wang and colleagues reported that 75.6% of participants (85.5% of women, 66.1% of men) of this study could correctly recognize depression in the case vignette [20]. The ability to recognize depression was associated with lower stigma, though this effect was more pronounced in women [20]. The results suggest that improving depression literacy may reduce stigma against depression. However, future large scale campaigns to promote depression should consider that depression literacy level in the population may be high. In our sample, the proportion of case recognition is 75.6%. Therefore, there could be a "ceiling effect" in promoting depression literacy at the population level. Nevertheless, the levels of mental health literacy can be varied by regions. In our sample, 24.4% could not recognize the depression case vignette. As such, some may argue that the negative attitudes of these individuals cannot be deemed as "stigma against depression." This view may be debatable because the case vignette depicts a person with major depression and the attitudinal scale was conceptualized as stigma against depression by the developers. To certain extent, people's views on the person in the case vignette can be considered their attitudes towards depression. The difference in the meanings of "stigma against depression" in different groups should be considered in interpreting the results.

Our study provided the descriptive information about stigma against depression in a Canadian general population sample, which can be used to assist in planning stigma reduction programs in Canada. For example, based on data from consecutive surveys about stigma, comparing changes in overall stigma scores can provide information about the effectiveness of stigma reduction programs at the population level - whether or not the programs have had positive impacts on stigma; comparing changes specific items would yield information about changes in specific areas.

This study has several limitations. First, while efforts were made to correct for the number of participants in each household, number of telephone lines, and the sex-age distribution of the province, it remained that only residential participants with a telephone were eligible for participation. The findings may be applicable to those who do not have telephone and those who are homeless. There existed the possibility for reporting and recall bias due to the study's reliance on self-reporting. Due to the cross-sectional nature of this study, only a correlation, and not a causal relationship, between variables can be established. Lastly, given the sample of this study was drawn from the Province of Alberta, it may not be possible to generalize this study to other populations.

## Conclusions

Stigma against depression differs by gender, age, education, and immigration status. The findings that men reported more stigmatizing attitudes than women, and that those with higher levels of education would report lower levels of depression stigma is consistent with existing literature. It was unexpected that immigration status would be so strongly related to levels of depression stigma, or that there would be no clear relationship between depression stigma and participant age. Future studies need to better understand the mechanisms underlying stigma against depression in the subpopulations so as to develop effective strategies to reduce stigma.

## Competing Interests

The authors declare that they have no competing interests.

## Authors' Contributions

In the preparation of this paper, TMC was responsible for literature review, data analysis, manuscript preparation and submission. JLW was involved in manuscript preparation and interpretation. Both authors read and approved the final manuscript.

## Pre-publication history

The pre-publication history for this paper can be accessed here:

http://www.biomedcentral.com/1471-244X/10/29/prepub
